# Mobile Phase Aging and Its Impact on Electrospray Ionization of Oligonucleotides

**DOI:** 10.1021/jasms.3c00264

**Published:** 2023-11-18

**Authors:** Guilherme J. Guimaraes, Jack G. Saad, Vidya Annavarapu, Michael G. Bartlett

**Affiliations:** † Department of Pharmaceutical and Biomedical Sciences, University of Georgia College of Pharmacy, Athens, Georgia 30602, United States; ‡ Micromeritics Instrument Company, 4356 Communications Drive, Norcross, Georgia 30093, United States

## Abstract

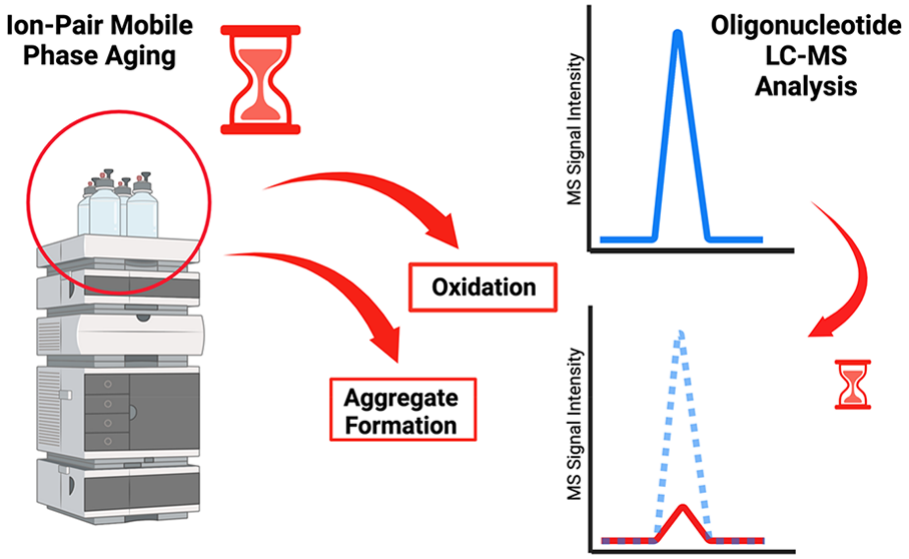

The implementation of fluoroalcohol/alkylamine mobile phase systems in oligonucleotide LC-MS provides a good balance between chromatographic separations and MS sensitivity. Since its introduction, several parameters including mobile phase composition, additive concentration, alkylamine hydrophobicity, and different fluoroalcohols have been carefully evaluated and optimized. While our understanding of this mobile phase system has increased over the years, there are challenges that continue to hinder method performance and remain poorly understood. One of these challenges is the constant loss of MS sensitivity over time, commonly termed mobile phase aging. This study investigates two aging mechanisms associated with loss of MS sensitivity: alkylamine oxidation and aggregate formation. The relationship between pH, organic solvent, oxygen, and mobile phase aging is characterized, and mitigation strategies to extend mobile phase lifetime are discussed.

## 1 Introduction

The unique combination between selectivity and sensitivity has made liquid chromatography–mass spectrometry (LC-MS) a popular analytical tool in the characterization of therapeutic oligonucleotides.^1,2^ LC-MS is versatile and can be used to support a wide variety of oligonucleotide analysis, including pharmacokinetic studies, sequencing of unknown strands, and secondary structure determination.^3−6^ LC-MS is typically chosen over hybridization assays to support these applications due to challenges with selectivity, normalization, and biased performance of different polymerase chain reaction (PCR) techniques.^3,7−10^

Within oligonucleotide chromatography, several techniques have been explored, including ion-pair reversed phase (IP-RP), hydrophilic interaction chromatography (HILIC), and anion exchange.^11−16^ Out of all these techniques and likely due to better compatibility with MS detection, IP-RP chromatography has been the predominant choice to support applications requiring additional MS sensitivity. However, method development can be challenging since chromatography optimization often has a negative impact on the MS signal. For example, the combination of alkylamines as ion-pairing agents and acetate as a counterion provides efficient separations but integrates poorly with mass spectrometry. Buffering mobile phases with formic acid or acetic acid causes ion suppression and limits method sensitivity.^17−19^ A common solution has been the replacement of acetate with fluoroalcohols.^20,21^ The alkylamine/fluoroalcohol mobile phase system supports desirable separations without sacrificing MS sensitivity, hence the popularity of this combination. This mobile phase system has been characterized in detail, and optimal parameters have been thoroughly investigated. The impact of additive concentration, hydrophobicity of the alkylamine, different fluoroalcohols, temperature, and organic modifier on method performance are well understood.^17,22−25^

While the alkylamine/fluoroalcohol mobile phase combination provides a good balance between chromatographic performance and electrospray sensitivity, there are disadvantages associated with its use. It is well-known that ion-pairs contaminate LC-MS systems. Constant source cleaning is required, while sensitive analysis requires the entire LC-MS system to be dedicated to oligonucleotide analysis. Another well-known limitation is the loss of MS sensitivity over short time periods, which is commonly termed mobile phase aging. Over 50% loss in sensitivity has been reported in as little as 24 h in an alkylamine/fluoroalcohol mobile phases system.^26^ Mobile phase aging is a known phenomenon, but the underlying reasons and mitigation strategies are scarce in the literature.

The most common mitigation strategy is to make mobile phases fresh daily, which does not align well with extended sequence runs from large sample batches and generates a larger waste of MS grade additives. Recently, a unique mitigation strategy has been reported by Li and co-workers.^10^ The study adopts the use of a ternary pump that prevents ion-pairs from sitting in water as mobile phases age in HPLC bottles. Mobile phase lifetime was extended substantially by aging in an organic solvent. However, many LC-MS systems use a binary pump design that does not support this application. To the best of our knowledge, the only additional study investigating mobile phase lifetime has been published by Li and co-workers.^27^ When the chromatographic performance of several alkylamines was evaluated, the formation of aggregates was detected by several analytical methods. It was hypothesized that aggregate formation could be linked to losses in MS sensitivity.

To better understand factors influencing mobile phase aging, this study investigates several parameters and their impacts on mobile phase lifetime. The relationship between pH, organic phase composition, and rate of mobile phase aging are reported. Lastly, we investigate two potential mechanisms influencing mobile phase lifetime: alkylamine oxidation and aggregate formation. Understanding aging mechanisms is paramount to developing new mitigation strategies to address the detrimental effects of mobile phase aging in oligonucleotide IP-RP LC-MS methods.

## 2 Materials and Methods

### 2.1 Materials

LC-MS grade water, methanol, and acetonitrile were obtained from Millipore Sigma (St. Louis, MO). Triethylamine (TEA, ≥99%) and formic acid were obtained from Alfa Aesar (Thermo Fisher Scientific Whatman, MA). Hexylamine (HA, ≥99%), and 1,1,1,3,3,3-hexafluoro-2-propanol (≥99%) were obtained from Sigma-Aldrich (St. Louis, MO). Triethylamine *N*-oxide (≥95%) was obtained from Boc Sciences (Shirley, NY). Nuclease-free water and pipet tips were purchased from Ambion (Thermo Fisher Scientific, Waltham, MA) and DNA Lobind microcentrifuge tubes were purchased from Eppendorf (Hauppauge, NY). The phosphorothioate oligonucleotide (T*C*C*G*T*C*A*T*C*G*C*T*C*C*A*G*G*G*G) was synthesized by Integrated DNA Technologies (Coralville, IA).

### 2.2 Sample Preparation

#### Oligonucleotide LC-MS Samples

For oligonucleotide samples, a 1 mg/mL stock solution was prepped in nuclease free water. From the 1 mg/mL solution, 10 or 100 μg/mL solutions were made in 10% methanol and stored in a –20 °C freezer. All oligonucleotide injections consisted of a 10 μg/mL oligonucleotide solution, except when the pH of mobile phases was buffered with formic acid, which used 100 μg/mL injections.

#### TEA and HFIP GC-MS samples

A stock solution of 285.5 mM HFIP and 211.25 mM TEA was prepared in methanol and used to make calibration curves of HFIP and TEA combined. The calibration curves for HFIP consisted of 4.46, 5.95, 8.93, 11.90, 17.85, 23.80, 35.70, 47.60, and 71.40 mM samples. The calibration curves for TEA consisted of 3.36, 4.48, 6.63, 8.96, 13.26, 17.93, 26.51, 35.85, and 53.03 mM samples in 80% MeOH. Prior to analysis, calibration curve samples were diluted 5-fold to account for a 5-fold sample dilution, ensuring that unknown samples were within range. Calibration curves for TEA and HFIP were analyzed with 1/X weight. The correlation coefficient for all calibration curves was greater than 0.99. QC concentrations can be seen in Supplemental Table 1.

Mobile phases aged in 10% methanol, 2 mL were removed from the bottle and diluted 5-fold for a final composition of 80% MeOH. 990 μL of the solution was combined with 10 μL of toluene (8.7 mg/mL) and added to a GC-MS vial.

Mobile phases aged in 100% methanol, 2 mL were removed from the bottle and diluted 5-fold for a final composition of 80% MeOH. 990 μL of the solution was combined with 10 μL of toluene (8.7 mg/mL) and added to a GC-MS vial.

### 2.3 LC-MS Instrumentation and Conditions

LC–MS experiments were performed by using a Waters Acquity Premier UPLC system coupled to a Waters Synapt G2 HDMS quadrupole time-of-flight hybrid mass spectrometer (Milford, MA). Tuning parameters were as follows: capillary voltage −2.0 kV, cone voltage 25 V, extraction cone voltage 2 V, source temperature 125 °C, desolvation temperature 450 °C, cone gas 0 L/h, and desolvation gas (nitrogen) 1000 L/h. The MS data acquisition was performed in negative-ion sensitivity mode with a 1 s scan time over the *m*/*z* range of 400–1200. Experiments illustrated in Figures 2–4 consisted of injections made through a union (without column) to make comparisons between mobile phase compositions more efficient. Different mobile phase compositions were pumped through line A at 100% A for 1.20 min per injection. Detailed mobile phase composition can be found in the figure caption.

For experiments illustrated in Figure 5, the union was replaced by a Waters Acquity UPLC PREMIER Oligonucleotide C18 column, 130 Å, 1.7 μm, 2.1 × 50 mm. Mobile phase A consisted of 15 mM TEA and 25 HFMIP in 10% methanol, and Mobile Phase B consisted of 100% MeOH. The gradient was as follows: 0% B from 0 to 1 min, 0–20% B from 1 to 5 min, and 0% B from 5.1 to 8 min. The column compartment was kept at 65 °C, and flow rate was 0.17 mL/min. Injection volume was 10 μL

Mobile phases stored in bottles with controlled air-flow used a Phenomenex SecurityCap closing system to minimize the amount of air coming into the bottles. Mobile phases stored in bottles without controlled air-flow consisted of traditional caps with openings that do not limit the amount of air coming in or out of the bottles

### 2.4 Dynamic Light Scattering Studies

Several mobile phase solutions were transferred to a 1 cm cuvette and placed in a Micromeritics NanoPlus HD dynamic light scattering system (Norcross, GA). The autocorrelation function (ACF) was monitored in all solutions for signs of Brownian motion indicated by an increase in the G_2_ (τ), which is indicative of particle-like behavior.

### 2.5 GC-MS Instrumentation and Conditions

A 6890 N gas chromatography system (Agilent, Santa Clara, CA, USA) equipped with a PAL autosampler (CTC Analytics, Zwingen, Switzerland) and a model 5973 single-quadrupole mass-selective detector with an electron ionization (EI) source (Agilent) was used for GC/MS analysis. Enhanced ChemStation software (Agilent) was used for instrument control and data processing. Chromatographic separation was achieved using a DB-624 GC column (60 m × 0.32 mm × 1.8 μm; Agilent) operating with a 1 mL/min constant flow of helium. The GC inlet was held at 240 °C. An injection volume of 1 μL was applied in split mode with a 25:1 split. The temperature program of the oven ran isothermally at 150 °C for 5.20 min. The transfer line was held at 220 °C. The ion source and quadrupole were held at 230 and 150 °C respectively. Full scans were acquired ranging from 50 to 200 amu. A 3.2 min solvent delay was applied to the method to protect the EI filament lifetime.

## 3 Results and Discussion

### 3.1 Ion-Pair Concentration in Mobile Phases over Time

Historically, the most popular mobile phase combination used in oligonucleotide IP LC-MS consists of TEA buffered with HFIP. Due to the volatility of both additives, their evaporation could be a potential cause of day-to-day MS signal variability. A GC-MS method was developed to further investigate TEA and HFIP concentrations in mobile phases capped with traditional LC bottle caps (which have additional openings to atmosphere). The precision and accuracy of the GC-MS method was validated according to the 2008 FDA bioanalytical method validation guidance and can be found in Supplemental Table 11. Toluene was used as an internal standard. During method development, two GC columns were evaluated: ZB-5MS and DB-264. The goal was to find a column where both analytes could be detected simultaneously. The best peak shape for triethylamine was obtained with the ZB-5MS column; however, poor retention and peak tailing was observed with HFIP. As an alternative, the method was validated using a DB-264 column, even though substantial tailing was observed for triethylamine (Supplemental Figure 1). Poor TEA peak shape did not impact precision and accuracy, therefore the method was validated and used to determine TEA and HFIP concentrations over time.

**Figure 1:**
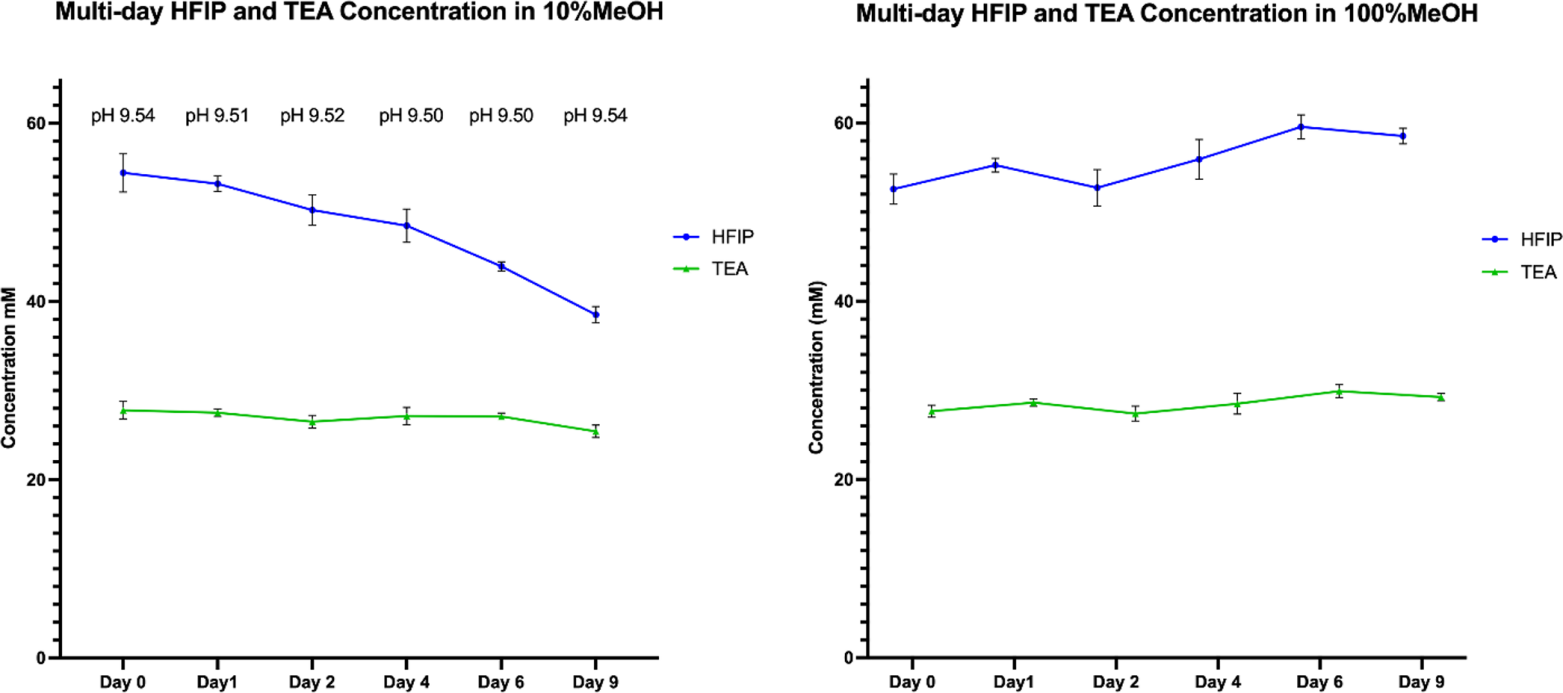
HFIP and TEA concentrations in 10% and 100% methanol aged in unsealed LC bottles.

Mobile phases containing 25 mM TEA and 55 mM HFIP in 10% methanol or 100% methanol were aged throughout a 9-day period. Figure 1 shows the concentration of HFIP and TEA over time in LC bottles containing 10% methanol or 100% methanol that were capped with traditional LC caps (open to atmosphere).

While TEA concentrations remained consistent, it is noticeable that HFIP evaporates at a considerable rate in the aqueous solution. However, no change in the mobile phase pH was observed. HFIP evaporation in aqueous solution was expected, given its low boiling point of 58 °C. In contrast, HFIP concentrations remained unchanged in 100% methanol. It is likely that improved solubility in methanol and methanol evaporation play a role in keeping HFIP concentrations consistent. As a control, the experiment was repeated with the same mobile phase composition (10% methanol and 100% methanol) kept in sealed bottles. No changes in TEA or HFIP concentrations were noticeable under those conditions (Supplemental Figure 2). While HFIP concentrations change over time, the slow rate of its evaporation does not justify the significant decrease in the MS signal, suggesting a more complex aging mechanism.

### 3.2 Amine Oxidation

**Figure 2:**
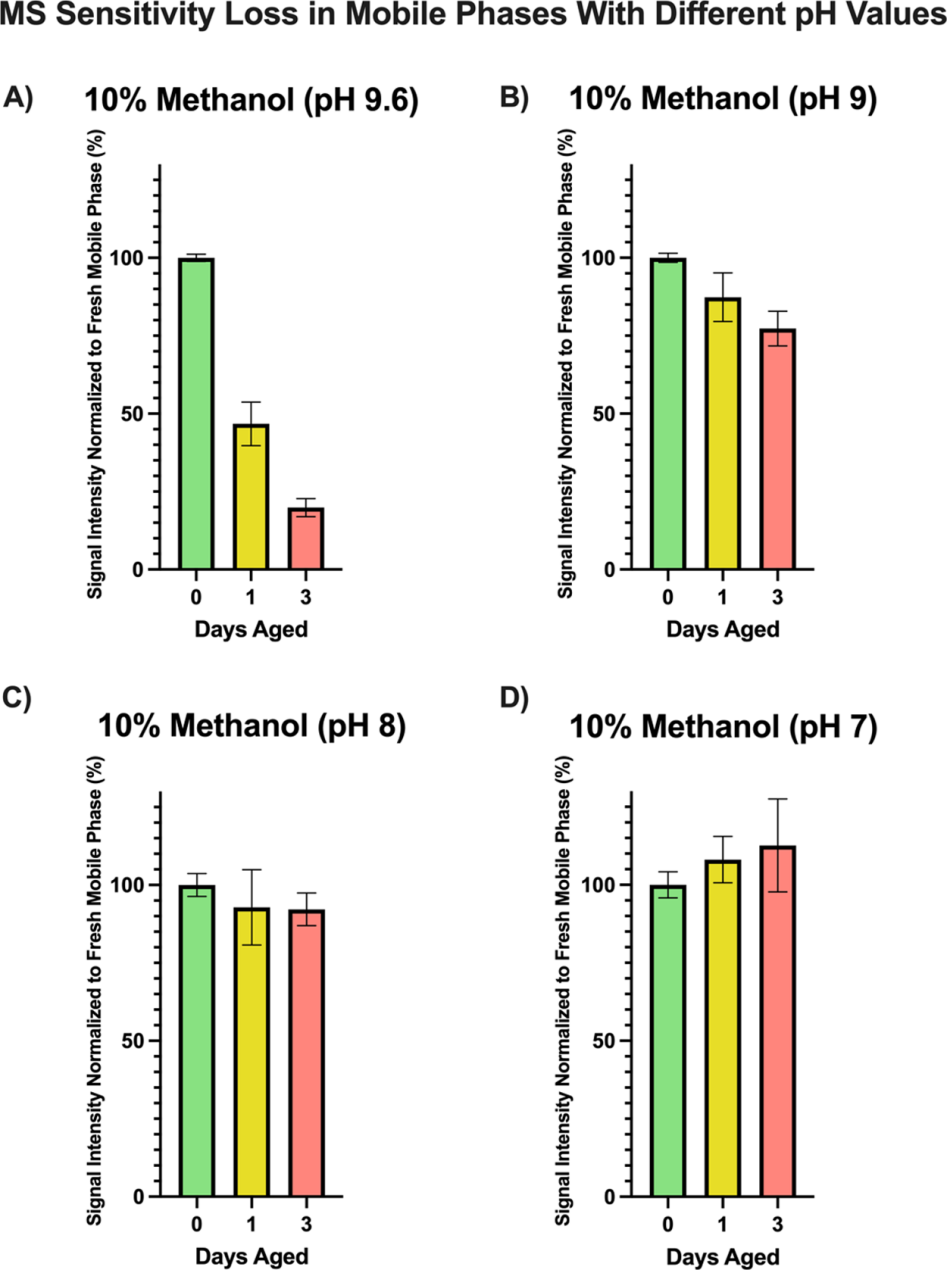
Daily variance in the electrospray sensitivity for mobile phases with different pH values. All mobile phases consist of 25 mM HFIP and 15 mM TEA in 10% MeOH with varying amounts of formic acid to reach different pH values: (A) pH 9.6; (B) pH 9; (C) pH 8; (D) pH 7. All values are normalized to the sensitivity obtained when mobile phases were made fresh. Sensitivity is shown as percentage to fresh mobile phases.

A potential mechanism for mobile phase aging could be associated with oxidation of the alkylamine. It has been previously reported that the rate in which TEA oxidizes is directly related to pH. Tao and co-workers report greater TEA oxidation rates under high pH conditions (pH 10) when compared to relatively lower pH conditions (pH 9, pH 8).^28^ To better understand the relationship between mobile phase pH and aging, we investigated the rate of mobile phase aging under 4 different pH conditions. Protonated amines decrease the rate of oxidation; therefore, MS signal loss from potential amine oxidation should decrease under lower pH values. Figure 2 shows losses of oligonucleotide MS signal over 1-day and 3-day time periods for mobile phase solutions buffered with formic acid to reach different pH conditions. The pH 9.6 represents the original pH of a mixture consisting of 25 mM HFIP and 15 mM TEA in 10% MeOH.

Under higher pH conditions (pH 9.6), there was a quick decrease in MS sensitivity after mobile phases aged for 1 and 3 days. The rate at which mobile phases age decreased as a function of pH, and aging was not noticeable at pH 7. These results suggest pH 7 to be the ideal condition, but there are special considerations to be made. The p*K*_a_ of TEA is 10.65, while the p*K*_a_ for HFIP is approximately 7.97. It is not possible to use HFIP as an acidic modifier to buffer mobile phases to pH 7. Different additives such as formic acid or acetic acid are needed, but they cause ion suppression in oligonucleotide IP LC-MS. Mobile phases with lower pHs will age at slower rates; however, signal intensity will be substantially lower from the beginning, due to the presence of formic acid or acetic acid.

**Figure 3:**
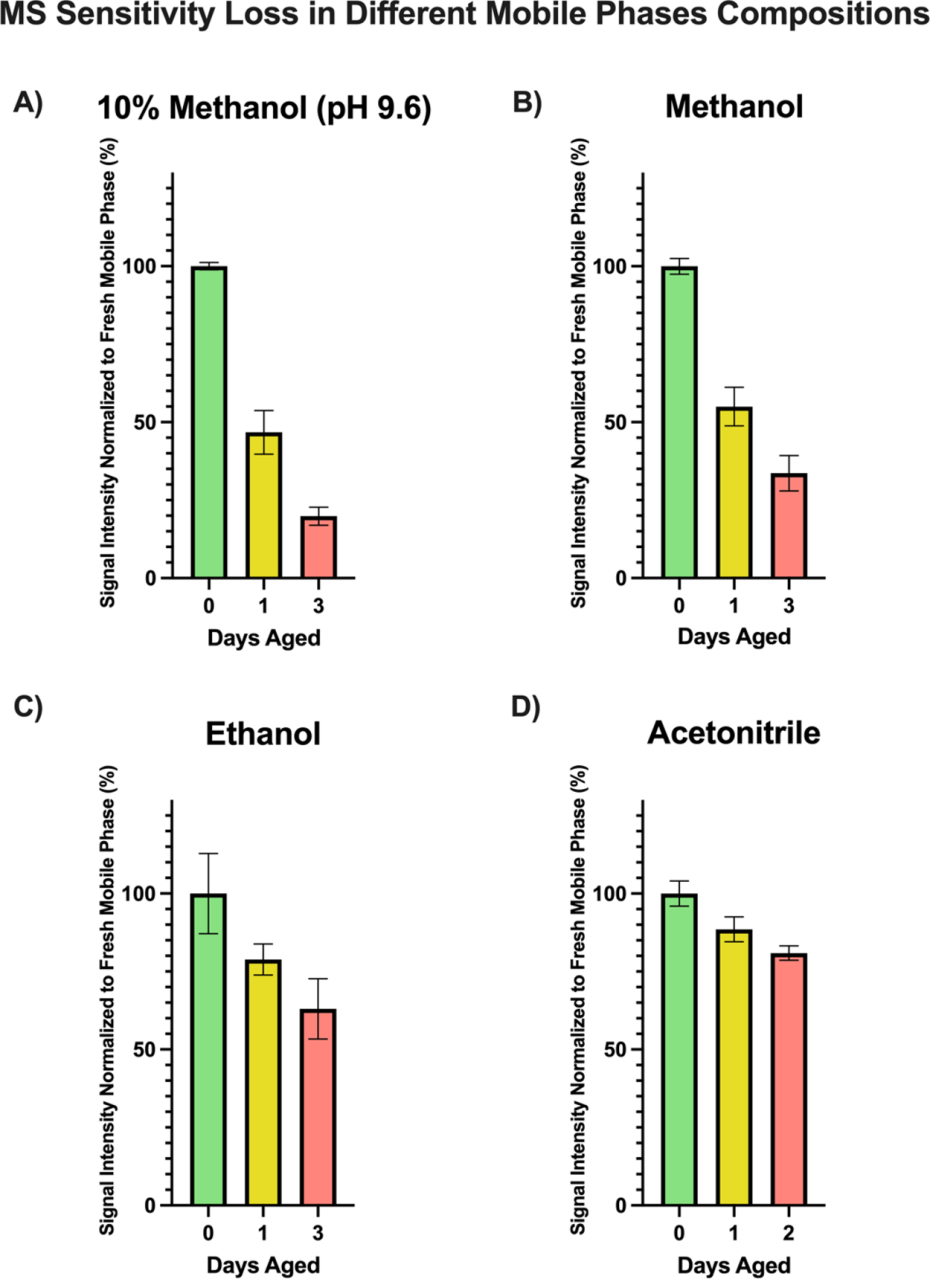
Daily variance in the electrospray sensitivity for mobile phases prepared in different solvents. Mobile phases consist of 25 mM HFIP and 15 mM TEA in (A) 10% methanol; (B) 100% methanol; (C) 100% ethanol; (D) 100% acetonitrile. All values are normalized to the sensitivity obtained when mobile phases were made fresh. Sensitivity is shown as percentage to fresh mobile phases.

In addition to pH, it is likely that a polar protic solvent medium would facilitate amine oxidation. Within popular solvents used in RP chromatography, the only polar aprotic solvent is acetonitrile. To test the hypothesis that aging should be slower in a polar aprotic medium, mobile phases were aged in 10% methanol (predominantly aqueous), methanol and ethanol (polar protic solvents), and in acetonitrile (polar aprotic solvent). Figure 3 shows losses of oligonucleotide MS signal over 1-day, and 3-day time periods of a 25 mM HFIP and 15 mM TEA solution prepped in different solvents.

As suspected, mobile phase aging was substantially decreased when ion-pairs were aged in a polar aprotic medium (acetonitrile). These observations help explain the success of a recent method published by Li and co-workers.^10^ The study developed a unique way to increase the mobile phase lifetime. Instead of using a traditional binary mobile phase system (with predominantly aqueous solution containing ion-pairs in line A and organic phase with ion-pairs in line B), the study implemented a ternary pump that removes ion-pairs from lines A and B and allows ion-pairs to sit in 100% acetonitrile (line C) instead of a protic medium. Line C is then used to deliver a constant flow of the ion-pairing solution. According to the study, removing ion-pairs from water increased the mobile phase lifetime substantially.

If amines are being oxidized, exposure to oxygen should either accelerate or decrease the rate at which mobile phases age. Rentel and co-workers have reported tributylamine to oxidize when exposed to air.^29^ Tributylamine oxide was shown to negatively impact the original method, so the authors recommend fresh tributylamine solutions to be stored under argon. Given the relationship between air exposure and amine oxidation, we used MS signal intensity to investigate how air exposure impacts method sensitivity. We investigated the rate in which mobile phases aged when bottles are sealed (limited contact with oxygen) and when bottles are open to atmosphere. As seen in Supplemental Figure 3, limiting the amount of oxygen slows down aging effectively.

**Figure 4:**
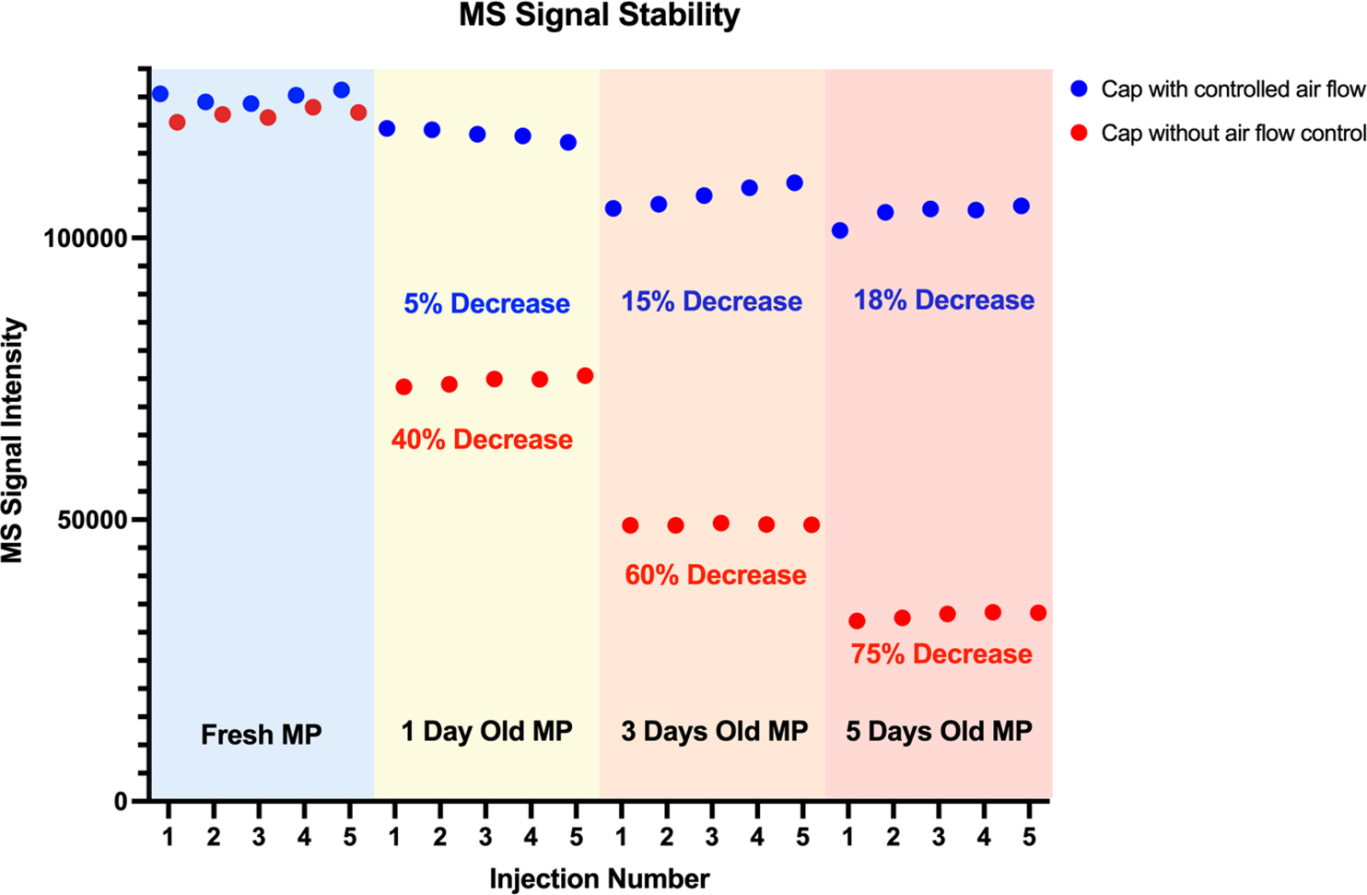
Electrospray sensitivity in mobile phases aged in bottles with (blue) or without (red) controlled airflow.

Mobile phase caps require oxygen flow in the bottles to allow solvents to be pumped into the LC system. Often times, these caps have openings for several lines, which, when not used, remain open to atmosphere and allow higher volumes of air to come in or out of bottles. More recent cap designs offer an “increased safety” feature by limiting the amount of air that comes out of bottles but also decreasing the amount of air that comes into the bottle as well, since unused cap openings are kept plugged and air flow is controlled by a filter apparatus. Given the slow aging shown in capped bottles, the simple solution of using caps that reduce air flow was investigated. Figure 4 shows mobile phase aging in bottles capped with traditional caps containing multiple openings and with caps that reduce air flow. Four time points were investigated: day 0 (fresh mobile phase), day 1, day 3, and day 5.

It is common for fresh mobile phases that do not contain ion-pairs to last for at least an entire week. As seen in Figure 4, a 75% decrease in signal intensity prevents alkylamines/fluoroalcohol mobile phases from being used in that manner. However, the simple solution of adopting caps that control airflow can dramatically increase mobile phase stability.

**Figure 5:**
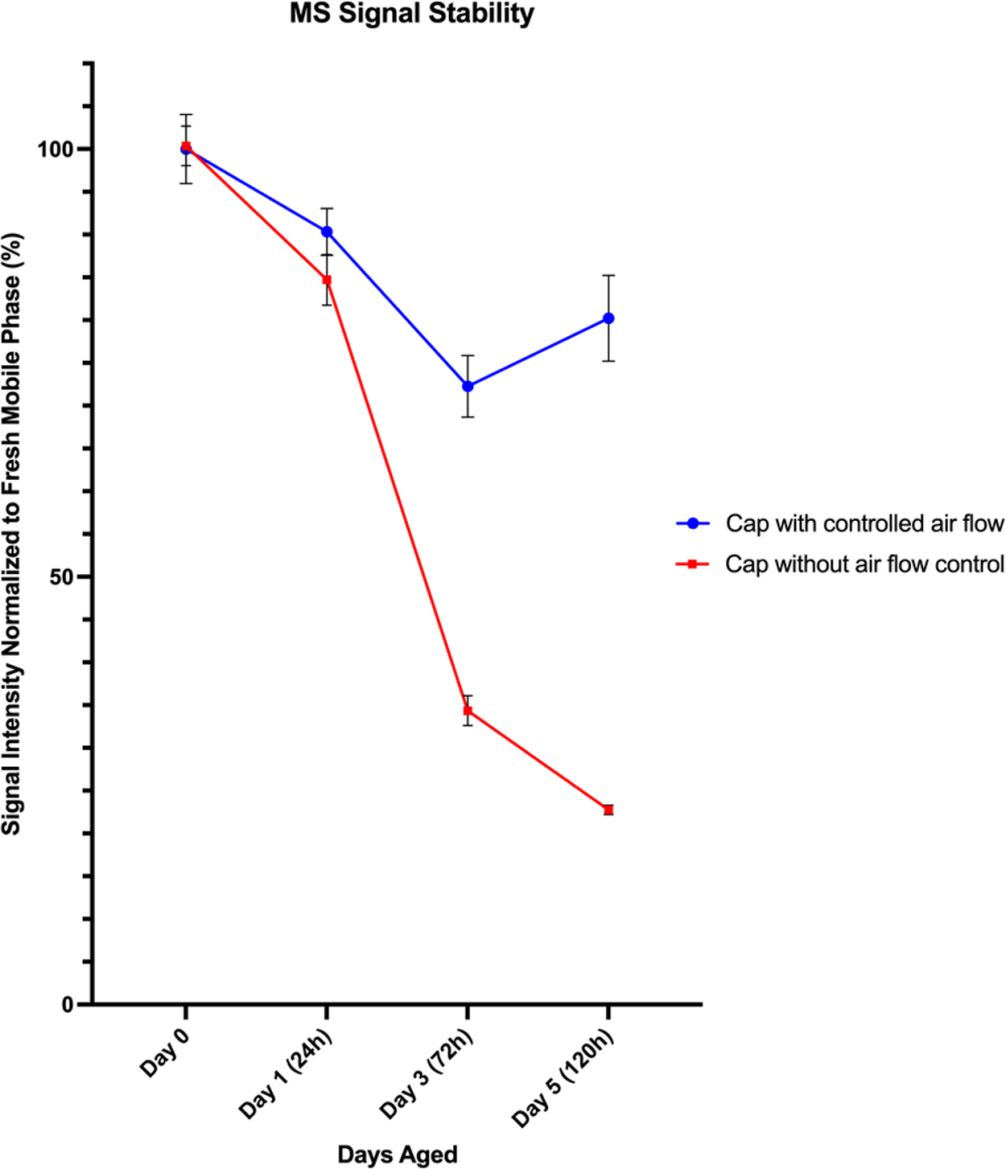
Electrospray sensitivity in mobile phases aged in bottles with (blue) or without (red) controlled airflow measured in column injections. Differences in day 3 and day 5 data points for Cap with “controlled air flow” were not statistically significant (*n* = 5, *p* > 0.05, paired *t* test).

Experiments presented in Figure 4 were repeated with the replacement of a union for an LC column. Mobiles phases with caps that control airflow and traditional caps were aged for 5 days. While injections through a union increased throughput and allowed the comparison of several different mobile phase compositions, injections in LC columns were still needed to ensure that the impact of mobile phase aging was still observed in a scenario in which chromatographic separations are taking place. Sensitivity was measured by taking the area under the curve for peaks obtained using fresh mobile phase and for peaks obtained once the same mobile phase was aged. As shown in Figure 5, similar sensitivity losses are seen in bottles with open air flow, while bottles with controlled air flow show good signal stability.

The relationship between rate of mobile phase aging, pH, aprotic solvent medium, and oxygen exposure strongly suggests that mobile phase aging is predominantly controlled by alkylamine oxidation. However, directly detecting amine oxidation was shown to be challenging. As triethylamine oxidizes, diethylamine and acetaldehyde should be forming. The stability of triethylamine in the GC experiments shown in Figure 1 suggests that degradation of TEA into DEA is not detectable in the mM level. Further into the oxidation mechanism, acetaldehyde could react with oxygen, forming paracetic acid, which could then react with triethylamine and form TEA *N*-oxide. Adding small concentrations (0.375 mM) of TEA *N*-oxide to freshly made mobile phases resulted in substantial losses of the original signal (Supplemental Figure 4). Artificial mobile phase aging with the addition of TEA *N*-oxide still supports the connection between mobile phase aging and amine oxidation; however, we were not able to measure TEA *N*-oxide in aged mobile phases. GC-MS experiments showed no presence of TEA *N*-oxide. Further investigation of the technique with injections of a TEA *N*-oxide standard showed thermal degradation of TEA *N*-oxide into TEA in the GC inlet. Thermal instability of N-oxide products in GC outlets have been previously shown for different analytes.^30^ Since GC was causing inlet degradation of TEA *N*-oxide, we attempted to use ESI to monitor N-oxide formation by switching the MS polarity to positive mode and monitoring the 118 *m*/*z* that is evident for TEA N-oxide formation. Infusing a TEA *N*-oxide standard (>95%) showed no in-source fragmentation with ESI, and the 118 *m*/*z* was able to be detected with no signs of triethylamine (*m*/*z* 102). An increase in the ratio of the TEA *N*-oxide *m*/*z* over the TEA *m*/*z* would provide evidence for oxidation. However, the ratio of 102 *m*/*z* to 118 *m*/*z* remained the same for fresh and aged mobile phases. While pH, −OH exposure, and oxygen exposure support amine oxidation, we were unable to validate this phenomenon quantitatively.

Reducing agents such as tris(2-carboxyethyl)phosphene (TCEP) have been previously added to mobile phases in small amounts to prevent degradation/oxidation of easily oxidizable analytes during HPLC analysis.^31^ Based on this report, trace amounts of TCEP (10 ppm) were added to mobile phases to prevent potential ion-pair oxidation. The benefit in the use of TCEP as an additive was not realized as substantial ion suppression was observed with the addition of TCEP in trace amounts (data not shown).

### 3.3 Aggregate Formation

Recent work by Li and co-workers have proposed the formation of aggregates in the alkylamine/fluoroalcohol mobile phase system as a potential aging mechanism.^27^ Autocorrelation functions derived from dynamic light scattering analysis of a mobile phase containing 65 mM DIEA and 50 mM HFIP in 10% methanol support the formation of aggregates. Furthermore, a direct correlation between aggregate formation and temperature was observed, where a higher rate of aggregate formation was observed under higher temperatures. Similar observations have been reported for nonionic detergents, where the critical micelle concentration (CMC) was decreased substantially as temperature increased from room temperature to 55 °C.^32^ Lastly, transmission electron microscopy images of the same mobile phase showed the physical formation of these aggregates. While the formation of aggregates was detected by several analytical methods, it is unknown if aggregate formation and loss of MS sensitivity are directly related.

**Figure 6:**
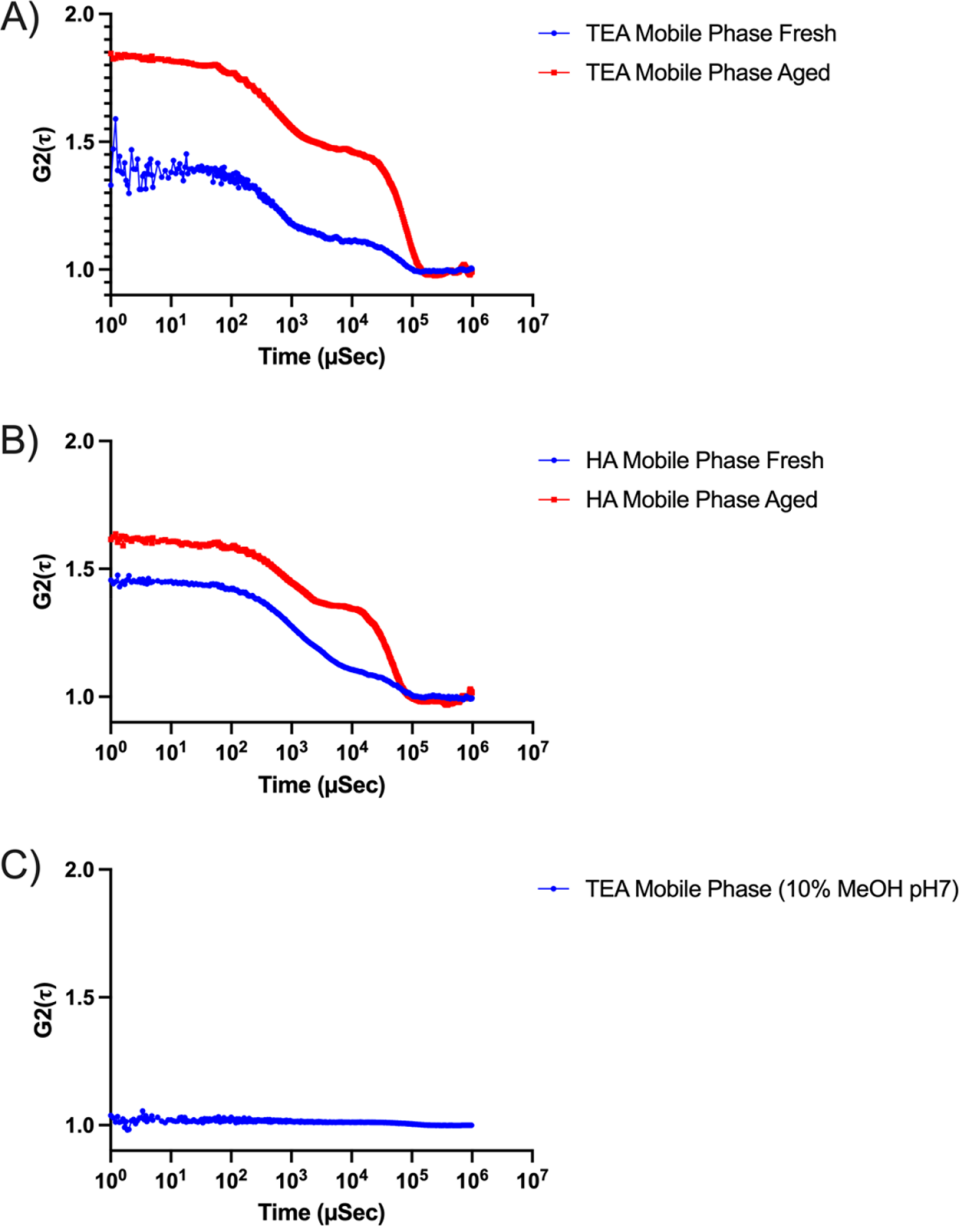
Autocorrelation function of (A) mobile phase consisting of 15 mM TEA and 25 mM HFIP in 10% methanol made fresh (blue) and aged for 5 days (red); (b) mobile phase consisting of 15 mM HA and 25 mM HFIP in 10% methanol made fresh (blue), and aged for 5 days (red); (C) mobile phase consisting of 15 mM TEA and 25 mM HFIP in 10% methanol buffered to pH 7.

The ability of alkylamines to form alkylamine micellar aggregates as well as mixed cationic surfactant/alkylamine micellar aggregates in aqueous solution has been previously characterized.^33,34^ Given the existing evidence for aggregates in this mobile phase system, we performed DLS experiments to characterize the relationship between aggregate formation and time. Mobile phases consisting of 15 mM TEA and 25 mM HFIP in 10% methanol were aged for 5 days and compared to freshly made mobile phases (Figure 6A). The same experiment was repeated for hexylamine (HA) 15 mM HA and 25 mM HFIP in 10% methanol (Figure 6B). Lastly, we investigated aggregate formation of a mobile phase consisting of 15 mM TEA and 25 mM HFIP in 10% methanol with the pH buffered to 7 as a negative control (Figure 6C), since it has been shown that an increase in the number of protonated amines may destabilize micellar aggregates, resulting in an increase in the CMC value and therefore no aggregate formation.^33^

As seen in Figure 6A and B, the autocorrelation function in aged mobile phases shows a larger presence of aggregates in aged mobile phases when compared to freshly made mobile phases. Higher S/N ratios in aged mobile phases indicate a direct relationship between time and the amount of aggregates being formed. The flat autocorrelation curve from Figure 6C suggests the absence of aggregates as amines become more protonated.

It has been previously reported that the composition of the aqueous medium is important in determining CMC. An increase in the CMC for sodium dodecyl sulfate was observed with an increase in the volume fraction of methanol in water.^35^ In agreement with this observation, DLS experiments performed in mobile phases consisting of 15 mM TEA in 100% methanol showed a flat autocorrelation curve with no aggregates being detected (Supplemental Figure 5). Even though no aggregates are observed in 100% methanol, a loss of MS sensitivity was still observed when mobile phases aged in 100% methanol (Figure 3B). Together, these findings suggest that the correlation between aggregate formation and loss in MS sensitivity is unlikely.

## 4 Conclusions

Lack of MS signal robustness remains a challenge in oligonucleotide IP-RP LC-MS analysis. The investigation of ion-pair concentration in solutions over time, pH, protic or aprotic medium, and its relationship to the loss of MS signal suggests alkylamine oxidation to be the most likely cause. However, direct measurements of oxidation byproducts were challenging and were unable to be completed. Aggregate formation, a potential aging mechanism that remains poorly understood, was further investigated. Based on DLS results, aggregate formation was detected; however, its connection to loss of MS signal is unlikely. Lastly, an alternative mitigation strategy to increase the mobile phase lifetime was introduced through the use of caps that control airflow.

## Supporting Information Available

Accuracy and precision for the GC-MS method; GC-MS chromatogram of HFIP, TEA and toluene; HFIP and TEA concentrations in 10% MeOH and 100% MeOH aged in LC bottles that were sealed; daily variance in electrospray sensitivity for mobile phases aged in LC bottles; MS signal intensity in fresh mobile phases; autocorrelation functions (PDF)

## References

[ref1] Sutton J. M.; Guimaraes G. J.; Annavarapu V.; van Dongen W. D.; Bartlett M. G. Current State of Oligonucleotide Characterization Using Liquid Chromatography-Mass Spectrometry: Insight into Critical Issues. J. Am. Soc. Mass Spectrom. 2020, 31 (9), 1775–1782. 10.1021/jasms.0c00179. 32812756

[ref2] Sutton J. M.; Kim J.; El Zahar N. M.; Bartlett M. G. Bioanalysis and Biotransformation of Oligonucleotide Therapeutics by Liquid Chromatography-Mass Spectrometry. Mass Spectrom. Rev. 2021, 40 (4), 334–358. 10.1002/mas.21641. 32588492

[ref3] Basiri B.; Sutton J. M.; Hooshfar S.; Byrnes C. C.; Murph M. M.; Bartlett M. G. Direct identification of microribonucleic acid miR-451 from plasma using liquid chromatography mass spectrometry. Journal of Chromatography A 2019, 1584, 97–105. 10.1016/j.chroma.2018.11.029. 30502920

[ref4] Sutton J. M.; El Zahar N. M.; Bartlett M. G. Oligonucleotide Anion Adduct Formation Using Negative Ion Electrospray Ion-Mobility Mass Spectrometry. J. Am. Soc. Mass Spectrom. 2021, 32 (2), 497–508. 10.1021/jasms.0c00380. 33476148

[ref5] Yuan L.; Dupuis J. F.; Mekhssian K. A Novel Hybridization LC-MS/MS Methodology for Quantification of siRNA in Plasma, CSF and Tissue Samples. Molecules 2023, 28 (4), 1618 10.3390/molecules28041618. 36838605 PMC9967190

[ref6] Jiang T.; Yu N.; Kim J.; Murgo J.-R.; Kissai M.; Ravichandran K.; Miracco E. J.; Presnyak V.; Hua S. Oligonucleotide Sequence Mapping of Large Therapeutic mRNAs via Parallel Ribonuclease Digestions and LC-MS/MS. Anal. Chem. 2019, 91 (13), 8500–8506. 10.1021/acs.analchem.9b01664. 31129964

[ref7] Gee H. E.; Buffa F. M.; Camps C.; Ramachandran A.; Leek R.; Taylor M.; Patil M.; Sheldon H.; Betts G.; Homer J.; West C.; Ragoussis J.; Harris A. L. The small-nucleolar RNAs commonly used for microRNA normalisation correlate with tumour pathology and prognosis. Br. J. Cancer 2011, 104 (7), 1168–1177. 10.1038/sj.bjc.6606076. 21407217 PMC3068486

[ref8] Wang L.; Meng M.; Reuschel S. Regulated bioanalysis of oligonucleotide therapeutics and biomarkers: qPCR versus chromatographic assays. Bioanalysis 2013, 5 (22), 2747–2751. 10.4155/bio.13.234. 24256356

[ref9] Miska E. A.; Alvarez-Saavedra E.; Townsend M.; Yoshii A.; Šestan N.; Rakic P.; Constantine-Paton M.; Horvitz H. R. Microarray analysis of microRNA expression in the developing mammalian brain. Genome Biology 2004, 5 (9), R68 10.1186/gb-2004-5-9-r68. 15345052 PMC522875

[ref10] Li P.; Gong Y.; Kim J.; Liu X.; Gilbert J.; Kerns H. M.; Groth R.; Rooney M. Hybridization Liquid Chromatography-Tandem Mass Spectrometry: An Alternative Bioanalytical Method for Antisense Oligonucleotide Quantitation in Plasma and Tissue Samples. Anal. Chem. 2020, 92 (15), 10548–10559. 10.1021/acs.analchem.0c01382. 32628461

[ref11] Lobue P. A.; Jora M.; Addepalli B.; Limbach P. A. Oligonucleotide analysis by hydrophilic interaction liquid chromatography-mass spectrometry in the absence of ion-pair reagents. J. Chromatogr A 2019, 1595, 39–48. 10.1016/j.chroma.2019.02.016. 30772056 PMC6500481

[ref12] Gilar M.; Fountain K. J.; Budman Y.; Neue U. D.; Yardley K. R.; Rainville P. D.; Russell R. J. II; Gebler J. C. Ion-pair reversed-phase high-performance liquid chromatography analysis of oligonucleotides:: Retention prediction. Journal of Chromatography A 2002, 958 (1), 167–182. 10.1016/S0021-9673(02)00306-0. 12134814

[ref13] Enmark M.; Harun S.; Samuelsson J.; Örnskov E.; Thunberg L.; Dahlén A.; Fornstedt T. Selectivity limits of and opportunities for ion pair chromatographic separation of oligonucleotides. Journal of Chromatography A 2021, 1651, 462269 10.1016/j.chroma.2021.462269. 34102400

[ref14] Roussis S. G.; Rentel C. Separation of phosphorothioate oligonucleotide impurities by WAX HPLC under high organic content elution conditions. Anal. Biochem. 2022, 659, 114956 10.1016/j.ab.2022.114956. 36270331

[ref15] Fornstedt T.; Enmark M. Separation of therapeutic oligonucleotides using ion-pair reversed-phase chromatography based on fundamental separation science. Journal of Chromatography Open 2023, 3, 100079 10.1016/j.jcoa.2023.100079.

[ref16] MacNeill R.; Hutchinson T.; Acharya V.; Stromeyer R.; Ohorodnik S. An oligonucleotide bioanalytical LC-SRM methodology entirely liberated from ion-pairing. Bioanalysis 2019, 11 (12), 1155–1167. 10.4155/bio-2019-0031. 31241345

[ref17] Chen B.; Mason S. F.; Bartlett M. G. The Effect of Organic Modifiers on Electrospray Ionization Charge-State Distribution and Desorption Efficiency for Oligonucleotides. J. Am. Soc. Mass Spectrom. 2013, 24 (2), 257–264. 10.1007/s13361-012-0509-5. 23325666

[ref18] Muddiman D. C.; Cheng X.; Udseth H. R.; Smith R. D. Charge-state reduction with improved signal intensity of oligonucleotides in electrospray ionization mass spectrometry. J. Am. Soc. Mass Spectrom. 1996, 7 (8), 697–706. 10.1016/1044-0305(96)80516-2. 24203563

[ref19] Huber C. G.; Krajete A. Analysis of Nucleic Acids by Capillary Ion-Pair Reversed-Phase HPLC Coupled to Negative-Ion Electrospray Ionization Mass Spectrometry. Anal. Chem. 1999, 71 (17), 3730–3739. 10.1021/ac990378j. 21662880

[ref20] Apffel A.; Chakel J. A.; Fischer S.; Lichtenwalter K.; Hancock W. S. Analysis of Oligonucleotides by HPLC-Electrospray Ionization Mass Spectrometry. Anal. Chem. 1997, 69 (7), 1320–1325. 10.1021/ac960916h. 21639339

[ref21] Apffel A.; Chakel J. A.; Fischer S.; Lichtenwalter K.; Hancock W. S. New procedure for the use of high-performance liquid chromatography-electrospray ionization mass spectrometry for the analysis of nucleotides and oligonucleotides. Journal of Chromatography A 1997, 777 (1), 3–21. 10.1016/S0021-9673(97)00256-2.

[ref22] Basiri B.; Murph M. M.; Bartlett M. G. Assessing the Interplay between the Physicochemical Parameters of Ion-Pairing Reagents and the Analyte Sequence on the Electrospray Desorption Process for Oligonucleotides. J. Am. Soc. Mass Spectrom. 2017, 28 (8), 1647–1656. 10.1007/s13361-017-1671-6. 28405940 PMC5569388

[ref23] Basiri B.; van Hattum H.; van Dongen W. D.; Murph M. M.; Bartlett M. G. The Role of Fluorinated Alcohols as Mobile Phase Modifiers for LC-MS Analysis of Oligonucleotides. J. Am. Soc. Mass Spectrom. 2017, 28 (1), 190–199. 10.1007/s13361-016-1500-3. 27644940 PMC5500909

[ref24] Kaczmarkiewicz A.; Nuckowski L.; Studzińska S.; Buszewski B. Analysis of Antisense Oligonucleotides and Their Metabolites with the Use of Ion Pair Reversed-Phase Liquid Chromatography Coupled with Mass Spectrometry. Critical Reviews in Analytical Chemistry 2019, 49 (3), 256–270. 10.1080/10408347.2018.1517034. 30612436

[ref25] Donegan M.; Nguyen J. M.; Gilar M. Effect of ion-pairing reagent hydrophobicity on liquid chromatography and mass spectrometry analysis of oligonucleotides. Journal of Chromatography A 2022, 1666, 462860 10.1016/j.chroma.2022.462860. 35123169

[ref26] Kim J.; El Zahar N. M.; Bartlett M. G. In vitro metabolism of 2′-ribose unmodified and modified phosphorothioate oligonucleotide therapeutics using liquid chromatography mass spectrometry. Biomedical Chromatography 2020, 34 (7), e4839 10.1002/bmc.4839. 32246854

[ref27] Li N.; El Zahar N. M.; Saad J. G.; van der Hage E. R. E.; Bartlett M. G. Alkylamine ion-pairing reagents and the chromatographic separation of oligonucleotides. Journal of Chromatography A 2018, 1580, 110–119. 10.1016/j.chroma.2018.10.040. 30409418

[ref28] Tao Y.; Liu T.; Yang X.; Murphy J. G. Kinetics and Products of the Aqueous Phase Oxidation of Triethylamine by OH. ACS Earth and Space Chemistry 2021, 5 (8), 1889–1895. 10.1021/acsearthspacechem.1c00162.

[ref29] Rentel C.; Gaus H.; Bradley K.; Luu N.; Kolkey K.; Mai B.; Madsen M.; Pearce M.; Bock B.; Capaldi D. Assay, Purity, and Impurity Profile of Phosphorothioate Oligonucleotide Therapeutics by Ion Pair-HPLC-MS. Nucleic Acid Therapeutics 2022, 32 (3), 206–220. 10.1089/nat.2021.0056. 35238617

[ref30] Wang P. P.; Bartlett M. G. Identification and Quantitation of Cocaine N-Oxide: A Thermally Labile Metabolite of Cocaine. Journal of Analytical Toxicology 1999, 23 (1), 62–66. 10.1093/jat/23.1.62. 10022211

[ref31] Weisel L.; Corcoran L.; Castro S.; He Y. A Robust HPLC Method for Easily Oxidizable Phosphine Ligand Analysis. Journal of Chromatographic Science 2023, bmad008 10.1093/chromsci/bmad008. 36828780

[ref32] Schick M. J. EFFECT OF TEMPERATURE ON THE CRITICAL MICELLE CONCENTRATION OF NONIONIC DETERGENTS. THERMODYNAMICS OF MICELLE FORMATION1. J. Phys. Chem. 1963, 67 (9), 1796–1799. 10.1021/j100803a013.

[ref33] Astray G.; Cid A.; Manso J. A.; Mejuto J. C.; Moldes O.; Morales J.; Quintás J. N-Alkylamines-Based Micelles Aggregation Number Determination by Fluorescence Techniques. J. Solution Chem. 2011, 40 (12), 2072–2081. 10.1007/s10953-011-9775-2.

[ref34] García-Río L.; Hervés P.; Leis J. R.; Mejuto J. C.; Rodríguez-Dafonte P. Reactive micelles: nitroso group transfer from N-methyl-N-nitroso-p-toluenesulfonamide to amphiphilic amines. J. Phys. Org. Chem. 2004, 17 (11), 1067–1072. 10.1002/poc.827.

[ref35] Niraula T. P.; Shah S. K.; Chatterjee S. K.; Bhattarai A. Effect of methanol on the surface tension and viscosity of sodiumdodecyl sulfate (SDS) in aqueous medium at 298.15–323.15 K. Karbala International Journal of Modern Science 2018, 4 (1), 26–34. 10.1016/j.kijoms.2017.10.004.

