# Supplementary material for: Mobile Phase Aging and Its Impact on Electrospray Ionization of Oligonucleotides
Source: J Am Soc Mass Spectrom. 2023 Nov 18;34(12):2691–9. doi: 10.1021/jasms.3c00264 (PMC10704597; doi:10.1021/jasms.3c00264)
Supplement: Supplementary file 1 — js3c00264_si_001.pdf [file js3c00264_si_001.pdf]

Supporting Information

Mobile Phase Aging and Its Impact on Electrospray Ionization of Oligonucleotides

Guilherme J. Guimaraes[1], Jack G. Saad[2], Vidya Annavarapu[1], Michael G. Bartlett*[1]

[1] Department of Pharmaceutical and Biomedical Sciences, University of Georgia College of Pharmacy, Athens, Georgia 30602, United States

[2] Micromeritics Instrument Company, 4356 Communications Drive, Norcross, GA, 30093, USA
* Corresponding Author - mgbart@uga.edu

Supplemental Table 1 - Accuracy and Precision for GC-MS Method

HFIP	Intra-day (n=5)			Inter-day (n=15)		
Nominal Concentration (mM)	Measured concentration ± SD (mM)	RSD (%)	RE (%)	Measured concentration ± SD (mM)	RSD (%)	RE (%)
4.46 (LLOQ)	4.57 ± 0.09	2.09	2.44	4.34 ± 0.27	6.08	2.77
8.93 (LQC)	9.61 ± 0.11	1.18	7.71	9.25 ± 0.35	3.98	3.59
21.42 (MQC)	23.40 ± 0.10	0.45	9.23	21.88 ± 1.34	6.25	2.14
57.12 (HQC)	56.67 ± 0.11	0.19	0.79	55.43 ± 1.71	2.99	2.96
TEA	Intra-day (n=5)			Inter-day (n=15)		
Nominal Concentration (mM)	Measured concentration ± SD (mM)	RSD (%)	RE (%)	Measured concentration ± SD (mM)	RSD (%)	RE (%)
3.36 (LLOQ)	3.52 ± 0.29	8.63	4.89	3.23 ± 0.32	9.39	3.82
6.72 (LQC)	7.43 ± 0.28	4.23	10.57	7.10 ± 0.42	6.30	5.66
16.13 (MQC)	17.72 ± 0.33	2.05	9.85	16.46 ± 1.05	6.51	2.03
43.02 (HQC)	42.96 ± 0.43	1.01	0.14	41.77 ± 1.38	3.20	2.91

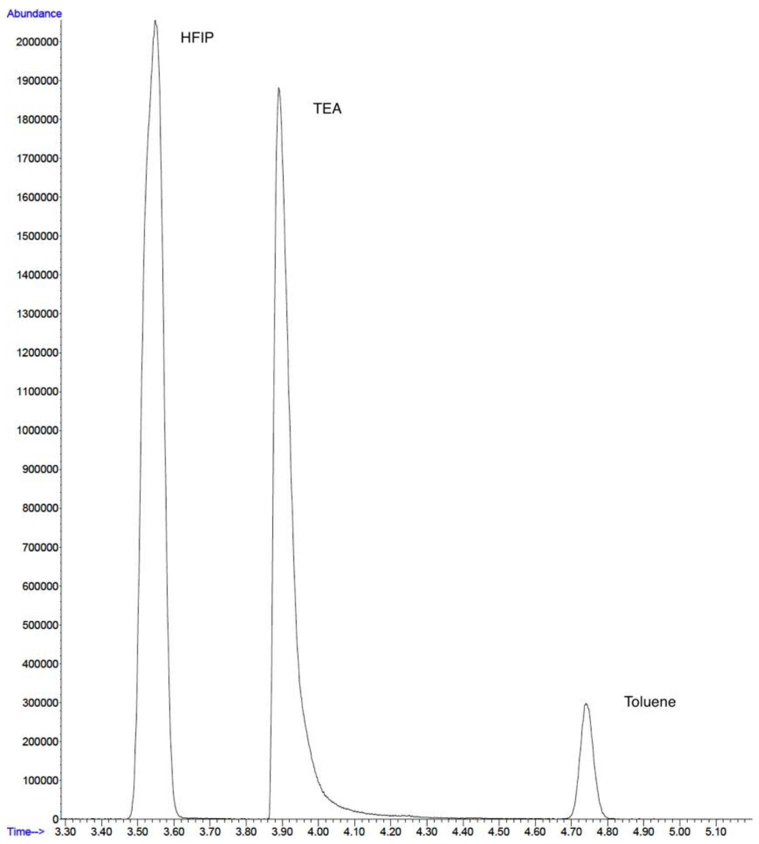

Supplemental Figure 1. GC-MS chromatogram of HFIP, TEA and toluene (IS).

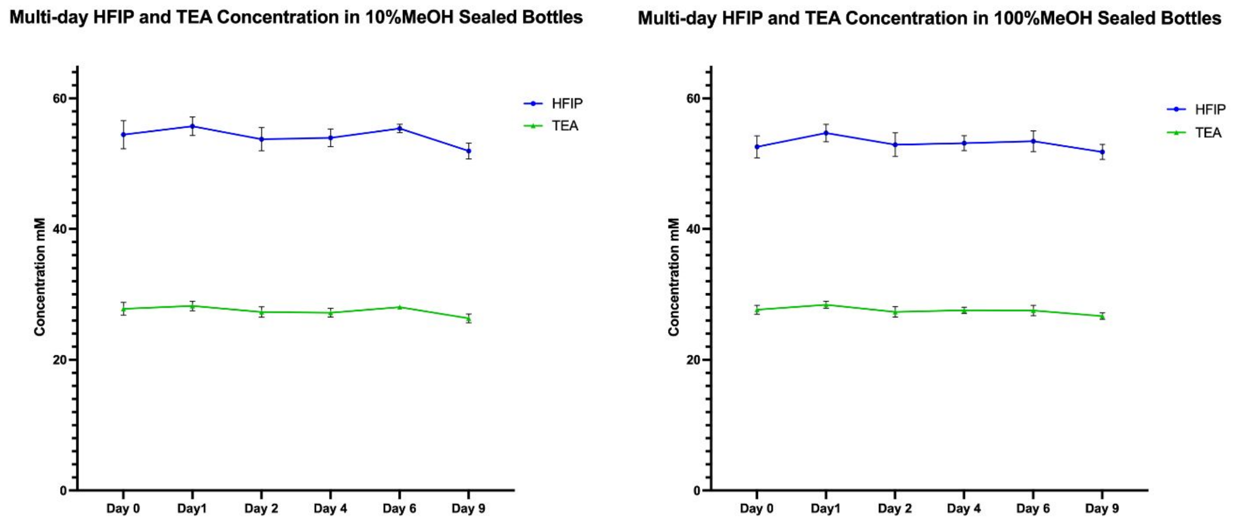

Supplemental Figure 2. HFIP and TEA concentrations in 10% MeOH and 100% MeOH aged in LC bottles that were sealed.

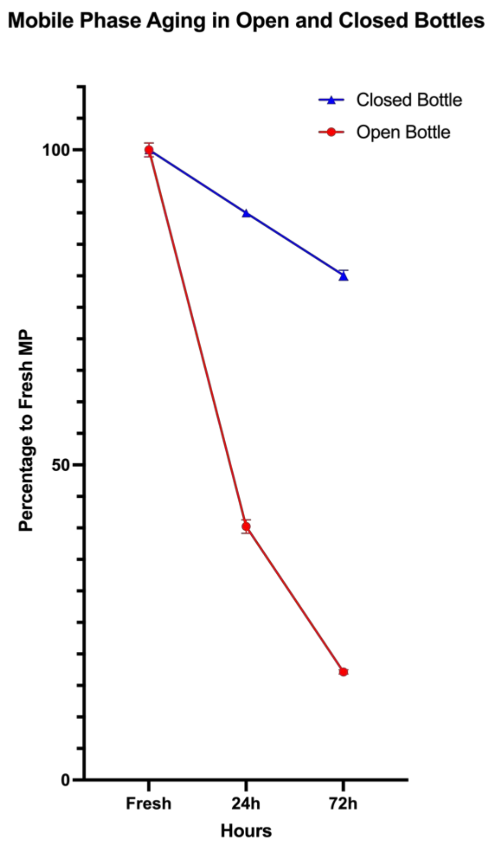

Supplemental Figure 3. Daily variance in electrospray sensitivity for mobile phases aged in LC bottles that were kept open (red) or bottles that were kept closed (blue).

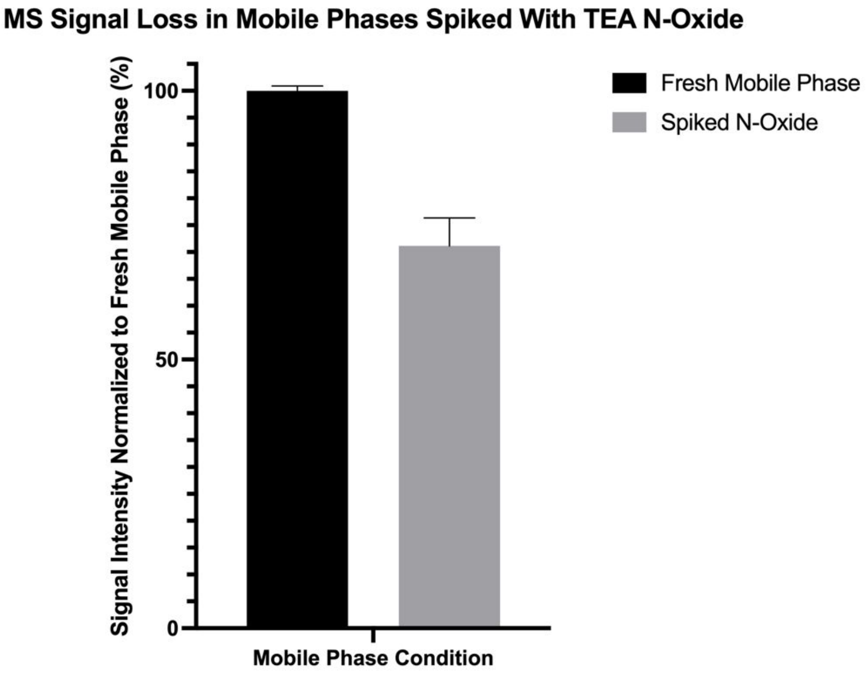

Supplemental Figure 4. MS signal intensity in fresh mobile phases (black) and in fresh mobile phases spiked with TEA N-oxide.

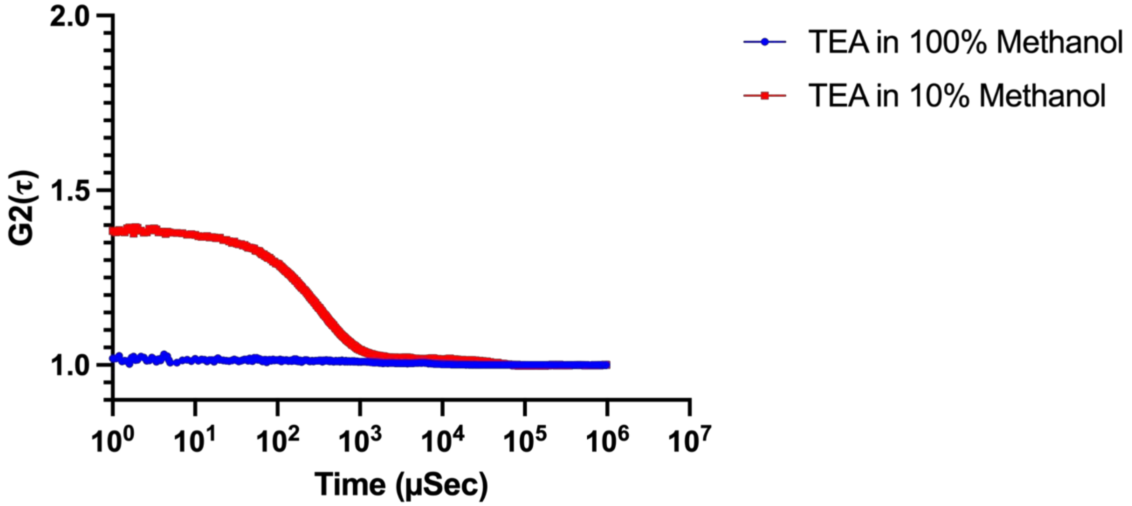

Supplemental Figure 5. Autocorrelation functions of a mobile phase consisting of 15mM TEA in 100% MeOH (red), and a mobile phase consisting of 15mM 10% MeOH (blue).
